# Effects of simulated nibbling intensity on growth and physiological characteristics of *Caragana tibetica*

**DOI:** 10.3389/fpls.2025.1625476

**Published:** 2025-09-23

**Authors:** Yumei Liang, Minghui He, Ruiting Jia, Qi Tian, Jiahuan Chen, Min Li

**Affiliations:** ^1^ Inner Mongolia Key Laboratory of Desert Ecological System, Inner Mongolia Academy of Forestry Sciences, Hohhot, Inner Mongolia, China; ^2^ College of Desert Control Science and Engineering, Inner Mongolia Agricultural University, Hohhot, Inner Mongolia, China; ^3^ Forest Resources Monitoring Office, Inner Mongolia Autonomous Region Forestry and Grassland Monitoring and Planning Institute, Hohhot, Inner Mongolia, China; ^4^ College of Computer Science, Inner Mongolia University, Hohhot, Inner Mongolia, China; ^5^ Northern Regional Office, Beijing Jianghe Huiyuan Technology Co., Ltd., Beijing, China

**Keywords:** *Caragana tibetica*, simulated gnawing, physiological characteristics, windbreak and sand fixation, desert grassland

## Abstract

As one of the typical sand-fixation and soil and water conservation shrub species in the desert steppe, the effects of simulated nibbling intensity on the growth and physiological characteristics of *Caragana tibetica* are unclear. To solve the problem, in this paper, *Caragana tibetica* with complete configuration, good growth status, close location, the same terrain, and basically consistent morphological structure in the test area were selected as the research objects. The artificial pruning method was adopted to simulate different intensities of animal nibbling. The field *in-situ* sampling and indoor index determination methods were used to determine its growth and physiological characteristics, so as to clarify the growth and physiological response characteristics of *Caragana tibetica* to simulated nibbling. The results showed that: (1) The higher the nibbling intensity, the greater the number of new branches and the length of new branches. (2) Different simulated nibbling intensities had significant effects on the physiological characteristics of *Caragana tibetica*, whose effects are through antioxidant enzymes, osmoregulatory substances, and malondialdehyde. (3) During the whole growing season of *Caragana tibetica*, the value of physiological characteristics increased first and then decreased, reaching the maximum value in July. (4) The growth characteristics and physiological characteristics of *Caragana tibetica* have different degrees of correlation. This paper clarifies the impact of simulated nibbling intensity on the growth and physiological characteristics of *Caragana tibetica*, which can provide an important theoretical basis and practical significance for promoting the sustainable development and utilization of the desert steppe.

## 1 Introduction

As an important part of China’s ecosystem, the desert steppe plays a crucial role in the construction of a green ecological security barrier in the north of China, the maintenance of terrestrial ecosystem functions, and sustainable development. Various shrub, semi-shrub, and small shrub communities in the desert steppe are not only precious biological resources and natural feed bases for animal husbandry, but also the core function of the desert ecosystem, in which *Caragana* plants play an important role (Ma et al., 2008). *Caragana tibetica*, which has relatively strong drought resistance, is one of the typical sand-fixation and soil and water conservation shrub species in the desert steppe (Liu et al., 2024a). In vegetation zoning, the *Caragana tibetica* community is often regarded as an indicator species marking the transition from grassland to desert. However, it was found that, unlike the good condition of *Caragana tibetica* in the free range, the health status of the ones in the enclosed sample area decreased obviously, where the plants grew slowly and some died. Although enclosure is an effective measure to restore degraded grassland vegetation, the enclosure time should not be too long to avoid inhibiting plant regeneration and seedling formation, whose appropriate time and method should be determined according to the type of grassland and the degree of degradation. Therefore, it is necessary to find out how livestock nibbling or human disturbance affects the growth and physiology of *Caragana tibetica* in free grazing land, to determine the appropriate time and method for the enclosure of *Caragana tibetica*.

Only a few existing studies focus on *Caragana tibetica.* Liu et al. (2024a) examined eight *Caragana tibetica* communities using point pattern analysis to assess the spatial distribution pattern (SDP) and the influencing factors of *Caragana tibetica* scrubs. Han et al. (2024) investigated the relationship between soil physical & chemical properties and the diversity of bacteria and fungi throughout the evolutionary stages of the scrub root systems. Liu et al. (2024b) employed Illumina paired-end sequencing to investigate the genetic organization and structure of *Caragana tibetica* and *Caragana turkestanica*. He et al. (2024) revealed that the grazing process induces significant changes in the metabolic pathways of *Caragana tibetica* branches by analyzing 48 metabolomic profiles from *Caragana tibetica*. Song et al. (2015) performed HPLC-based activity profiling for targeted identification of anti-HCC activity from CT-EtOAc by MS-directed purification method. Zhang et al. (2011) studied whether fertile islands were present inside and underneath *Caragana tibetica*-formed nebkhas in the northwest portion of the Ordos on the Inner Mongolia Plateau, China, and if such a fertile island effect increased with the age of the nebkhas. Xiang et al. (2005) studied antioxidant constituents of *Caragana tibetica* and found that the 70% acetone extract of the stems of *Caragana tibetica* has a potent superoxide anion scavenging activity.

There are relatively many studies on *Caragana* species, where most scholars devote themselves to studying the physiological characteristics of *Caragana* species in terms of photosynthesis and water use efficiency. Yan et al. (2025) studied how high temperature, salinity, and CO_2_ affect endogenous phytohormones, photosynthesis, and redox homeostasis in *Caragana korshinskii Kom* leaves, as well as comprehensively evaluating the plant’s physiological response to multiple environmental stressors. Lu et al. (2024) calculated the instantaneous water use efficiency and short-term water use efficiency of these plantations to investigate the impact factors. Yan et al. (2023) evaluated the physiological responses of *Korshinsk peashrub* (*Caragana korshinskii Kom.*) to water deficit, photosynthetic gas exchange, chlorophyll fluorescence, and the levels of superoxide anion. Gao et al. (2023a) clarified the response of photosynthesis physiology of *Caragana* intermedia to soil water content and to determine the relevant threshold ranges in the sandy lands of Qinghai-Tibet Plateau. Zhao et al. (2021) studied water-use strategies and physiological characteristics of *Caragana korshinskii* and *Salix psammophila* in a semiarid revegetated ecosystem. Yang et al. (2020) studied photosynthesis and water potential of *Caragana* intermedia, and its adaptation to environmental changes while growing. Zheng et al. (2017) selected three *Caragana* species to investigate the cell water relations for these regional-level distribution characteristics. Bao et al. (2015) analyzed age-related changes in photosynthesis and water relations of revegetated *Caragana korshinskii* in the Tengger Desert, Northern China. Guo et al. (2014) compared the photosynthesis and antioxidative protection in *Sophora moorcroftiana* and *Caragana maximovicziana* under water stress. Yan et al. (2012) studied photosynthesis, PSII efficiency, contents of ions, and free amino acids in leaves of *Caragana korshinskii Kom* exposed to three levels of salinity. Ma et al, 2004, Ma et al, 2003) studied photosynthesis, transpiration, and water use efficiency of *Caragana microphylla*, *Caragana intermedia*, and *Caragana korshinskii*.

Some scholars also studied other physiological characteristics of *Caragana* species, including stress resistance, adaptability to water, nitrogen, and phosphorus, carbon sequestration ability, physiological adaptability, and the resulting species pattern distribution. Ma et al. (2023) evaluated the stress resistance ability by the roots exposure stress model test and compared the physiological growth indexes in *Hedysarum scoparium* and *Caragana korshinskii*. Roy et al. (2021) searched for suitable combinations of water, nitrogen, and phosphorus to improve physiological and biochemical adaptations of *Caragana korshinskii* to coal-mined spoils. Gong et al. (2016) used five experimental sites to evaluate the *Caragana korshinskii*’s functional and physiological features, particularly its carbon fixation capacity, as well as the relationships among these features. Ma et al. (2016) studied drought responses of three closely related *Caragana* species and the implications for their vicarious distribution. Xie et al. (2014) studied the relationships between the leaf ecological and physiological traits of *Caragana* species and the aridity index and solar radiation, to examine the mechanisms responsible for the *Caragana* species distribution pattern. Ma et al. (2012) proposed correlation analyses on the relationship between physiological and morphological traits and climatic factors (precipitation and solar radiation) of three *Caragana* species.

In summary, remarkable progress has been made in the study of the growth and physiological characteristics of *Caragana* species, which can provide important references for this study. However, the effects of different nibbling intensities on the growth and physiological characteristics of *Caragana tibetica* are still unclear. Therefore, in this study, *Caragana tibetica*, the main protective plant in the transition zone between steppe and desert, is taken as the research object. Comparative experiments are carried out to simulate different nibbling intensities, growth characteristics of new shoot number and new shoot length of *Caragana tibetica* after nibbling are analyzed, and the physiological response characteristics (including antioxidant enzyme activity, osmotic regulator, etc.) of *Caragana tibetica* are clarified. The relationship between physiological characteristics and growth indicators is also analyzed, which can provide an important theoretical basis and practical guiding significance for promoting the sustainable development and utilization of the desert steppe.

## 2 Materials and methods

### 2.1 Study area

The study area is located in the middle of the Darhan Muminggan United Banner, Baotou, Inner Mongolia, near the National Highway G210. The geographical location is 110°5’43” -110°6’ 42” east longitude and 42°1’41” -42°1’ 7” north latitude. The study area is deep in the hinterland, which is located in the middle temperate zone. It is a typical temperate semi-arid continental climate, whose daily temperature difference is large, and the average annual temperature is about 6.2°C. The maximum length from east to west is about 1.2 km, the maximum width from north to south is about 1.3 km, and the average altitude is 1376 m. The average annual precipitation in the study area is about 170 mm, and the average annual sunshine duration is 3105.1h. The main wind direction is southeast, and the average annual wind speed is 4.0m/s. Strong winds appear each year, while sandstorms, blowing sand, and hurricanes sometimes appear.

### 2.2 Experimental design and sample collection

The experimental samples were collected in mid-April 2023. *Caragana tibetica* with complete configuration, good growth conditions, same terrain, and consistent morphological structure was selected as the research object in the study area. Since the sites were all within a 200 m range, the geographical characteristics and meteorological factors were consistent. Based on previous investigations and research over the years, the study sites had similar vegetation and soil types and altitudes. The artificial pruning method was adopted to simulate animal nibbling of different intensities. According to the volume percentage, the aboveground parts of the *Caragana tibetica* were pruned by 25%, 50%, and 75%, respectively. The unpruned shrubbery was used as CK (i.e., the control group). 5 replicates were set in each group, with a total of 20 plants. The pruning intensity of each plant was marked and numbered with PVC (i.e., Poly Vinyl Chloride) pipe. The number and length of new branches after pruning were observed and recorded at the end of the growing season (September of the same year). Healthy *Caragana tibetica* leaves were collected in May, June, July, August, and September, respectively to determine plant physiological characteristics including POD, SOD, Catalase (CAT), PRO, SS, Soluble Protein (SP), and Malondialdehyde (MDA). The samples collected in each test were immediately stored in dry ice and transported to the laboratory.

### 2.3 Growth characteristics measurement

New branch number (NBN) and new branch length (NBL) are selected as growth characteristics of *Caragana tibetica*.

#### 2.3.1 NBN

To measure NBN, at the end of the growing season (September), the number of new branches per plant of CK, 25% treatment group, 50% treatment group and 75% treatment group were recorded respectively, and the average number of new branches for 5 *Caragana tibetica* plants in each treatment group was treated as NBN in this group.

#### 2.3.2 NBL

As for NBL, at the end of the growing season (September), the length of each new branch in the CK, the 25% treatment group, the 50% treatment group, and the 75% treatment group was measured by a vernier caliper (accuracy 0.01mm). The average length of all new branches in each plant was taken as the length of the new branch, and the average length of a new branch of 5 *Caragana tibetica* plants in each treatment group was taken as NBL in this group.

### 2.4 Physiological characteristics determination

#### 2.4.1 POD

POD activity was determined using the Guaiacol colorimetry method (Yoshida et al., 2003). Extraction of plant samples was combined with H_2_O_2_ and PBA solutions, and absorbance at 470 nm was measured using a spectrophotometer to assess POD activity. Quantitative determination of POD is achieved by comparing the absorbance value to the standard curve of a standard sample.

#### 2.4.2 SOD

SOD activity was determined by Nitrogen blue tetrazole photochemical reduction method (Gao et al., 2023b). The sample extract was reacted with NBT solution, xanthine oxidase solution, NaH_2_PO_4_ solution, and Na_2_AsO_2_ solution under light conditions. When the reaction stops, the absorbance at 560 nm was measured using a spectrophotometer and quantified by calculating the SOD activity from a standard curve.

#### 2.4.3 CAT

CAT activity was determined by the Active ultraviolet absorption method (Mhamdi et al., 2010). The *Caragana tibetica* sample was diluted to a suspension, and the reaction mixture was prepared by mixing the diluted sample extract, phosphate buffer, KCoCl_2,_ and H_2_O_2_ in a test tube. Start the CAT to catalyze H_2_O_2_, and observe the formation of bubbles after the reaction stops. A spectrophotometer was used to measure absorbance at wavelengths between 240 and 290 nm to assess CAT activity. The absorbance data is compared with the standard curve for quantitative determination.

#### 2.4.4 PRO

PRO content was determined by the Sulfosalicylic acid extraction method (Ábrahám et al., 2010). After removing the precipitate by centrifugation, PRO was precipitated by trichloroacetic acid and redissolved. Using the reaction of diethylamine reagent at 60°C, the PRO content was calculated according to the standard curve.

#### 2.4.5 SS

SS content was determined by the Anthrone colorimetric method (Somani et al., 1987). *Caragana tibetica* samples were ground into powder and placed in a 50 mL centrifuge tube first. Then, adding an appropriate amount of distilled water, a boiling water bath is used to extract SS. After the insoluble matter was removed by centrifugation, the supernatant was reacted with anthrone reagent, and the color was developed in a boiling water bath. After cooling, the absorbance was finally measured at 620 nm using a spectrophotometer, and the SS content in the test sample was calculated according to the standard curve.

#### 2.4.6 SP

SP content was determined using the Coomassie brilliant blue method (Deans et al., 2018). First of all, *Caragana tibetica* samples were chopped and placed in a 50 mL centrifuge tube. Subsequently, 10 mL of Tris-HCl buffer was added, and the sample was dispersed using an ultrasonic instrument. After the cell fragments were removed by centrifugation, different concentrations of BSA standard and Bradford reagent were added to the standard and the test sample tubes, and the reaction time was 25 min. Finally, the absorbance was measured at 595 nm using a spectrophotometer, and the protein concentration in the test sample was calculated based on the standard curve.

#### 2.4.7 MDA

MDA content was determined by the Thiobarbituric acid method (Davey et al., 2005). *Caragana tibetica* samples were heated in a phosphoric acid buffer, and the absorbance at 532 nm was measured using a spectrophotometer. MDA content is calculated according to standard curves.

### 2.5 Data processing and analysis

Differences between the groups for the given parameters were evaluated using Duncan’s significance difference test, with significance set at *p*<0.05. The correlation analysis between the growth characteristics and physiological characteristics of *Caragana tibetica* after simulated eating was carried out with the *pheatmap* package in R 4.3.2 software, to determine the key factors affecting the growth characteristics and physiological characteristics.

## 3 Results

### 3.1 Changes in the NBN and NBL of *Caragana tibetica* under different simulated nibbling intensities

As shown in **Table 1**, NBN and NBL under different simulated nibbling intensities show significant differences (*p*<0.001). With the increase of nibbling intensity, both NBN and NBL showed a significant increasing trend, and both reached the maximum value at 75% nibbling intensity. It can be calculated that NBN and NBL under three kinds of nibbling intensity were higher than those in the control group (i.e. CK), where the ones at 75% nibbling intensity were 11.00 times and 7.86 times of CK, the ones at 50% nibbling intensity were 5.00 times and 3.61 times of CK, the ones at 25% nibbling intensity were 2.00 times and 1.81 times of CK. There were no significant differences in the NBN and NBL at CK and 25% nibbling intensity (*p*>0.05), while there were significant differences at 50% and 75% nibbling intensity (*p*<0.05), which proves that nibbling can promote the growth of new branches of *Caragana tibetica*.

**Table 1:** Changes in the NBN and NBL of *Caragana tibetica* under different simulated nibbling intensities.

Categories	Simulated nibbling intensities (Mean ± std.)				F	p
	CK	25%	50%	75%		
NBN/number	1.00 ± 0.84C	2.00 ± 0.84C	5.00 ± 1.64B	11.00 ± 2.49A	44.35	< 0.001
NBL/mm	1.40 ± 0.35c	2.54 ± 0.94c	5.06 ± 1.14b	11.00 ± 1.36a	88.14	< 0.001

Different uppercase letters and lowercase letters indicated that there were significant differences in NBN and NBL under different simulated nibbling intensities (*p*<0.05).

### 3.2 Changes of antioxidant enzyme activity in *Caragana tibetica* under different simulated nibbling intensities

The activity of the three antioxidant enzymes (i.e. POD, SOD, and CAT) was highest in July in the growing season (**Table 2**). POD content in *Caragana tibetica* leaves at CK, 25%, 50% and 75% nibbling intensity in July was 2.79 u•(g•min)^-1^, 2.91 u•(g•min)^-1^, 3.22 u•(g•min)^-1^ and 3.34 u•(g•min)^-1^, respectively. Compared with CK, 50% and 75% simulated nibbling intensity significantly increased by 15.41% and 19.71% (*p*<0.05). POD content was the lowest in May, when 75% nibbling intensity significantly increased by 32%, 8.55%, and 13.79% compared with CK, 25%, and 50% (*p*<0.05), respectively. It is clear that SOD content in July showed an increasing trend with the increase of nibbling intensity, when 25%, 50% and 75% nibbling intensity significantly increased by 14.39%, 21.43% and 35.27% compared with CK, respectively (*p*<0.05). However, there was no significant difference among the four treatment groups (CK, 25%, 50%, and 75%) in September (*p*>0.05). During the whole growing season, the CAT content of *Caragana tibetica* showed a trend of first increasing and then decreasing. There was no significant difference in CAT content between different treatment groups from May to August (*p*>0.05), while there was a significant difference between 50% and 75% nibbling intensity and CK in September (*p*<0.05).

**Table 2:** Changes of three types of antioxidant enzymes content in *Caragana tibetica* leaves under different simulated nibbling intensities.

Month	May				June				July				August				September			
SGI	CK	25%	50%	75%	CK	25%	50%	75%	CK	25%	50%	75%	CK	25%	50%	75%	CK	25%	50%	75%
POD/ u•(g•min)^-1^	1.25±0.05c	1.52±0.11b	1.45±0.05b	1.65±0.04a	1.85±0.1b	1.98±0.06b	2.14±0.09a	2.2±0.04a	2.79±0.19b	2.91±0.11b	3.22±0.1a	3.34±0.07a	2.5±0.06b	2.59±0.09b	2.51±0.13b	2.79±0.07a	1.5±0.07b	1.56±0.1b	1.6±0.06b	1.84±0.12a
SOD/ u•g^-1^	22.03±1.33c	25.17±1.59bc	27.09±3.2ab	29.62±0.76a	41.82±1.26c	43.34±1.7c	51.59±1.16b	56.23±2.63a	61.21±0.91d	70.02±1.3c	74.33±3.19b	82.8±2.29a	50.19±1.86c	49.95±2.21c	55.41±2.08b	61.37±1.1a	32.47±1.51a	35.47±1.13a	35.07±2.28a	35.13±1.49a
CAT/ mg•(g•min)^-1^	237.76±5.69a	244.36 ±4.22a	246.92±5.17a	253.05±14.29a	281.37±10.25a	287.01±14.05a	309.22±22.24a	316.49±14.46a	356.19±32.17a	381.73±13.31a	373.42±19.47a	415.26±15.27a	311.73±24.22a	325.96±28.24a	313.82±14.7a	336.34±21.89a	264.76±10.66b	283.49±16.18ab	295.31±7.89a	294.04±7.78a

Notes: Comparisons were made within the same month group under the same indicator, and there were no significant differences between data with the same lowercase letter (p<0.05). SGI represents simulated nibbling intensities.

**Figure 1:**
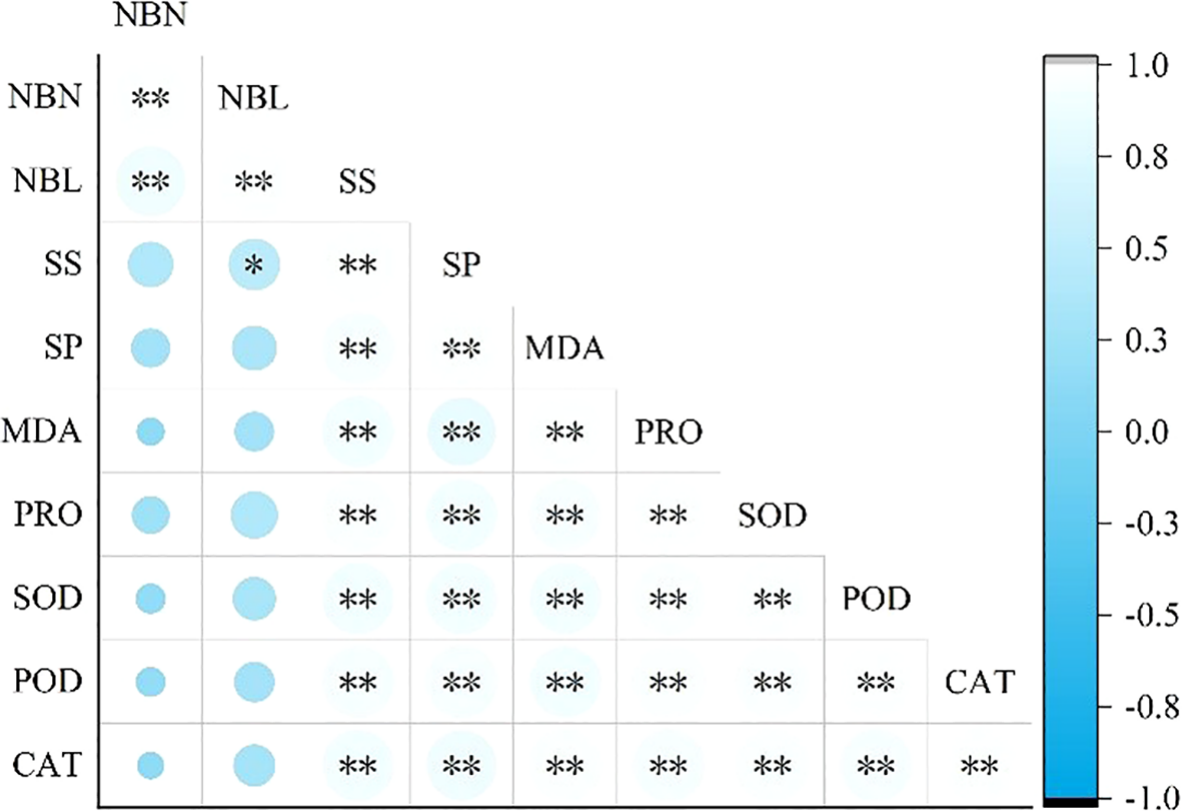
Correlation between growth and physiological characteristics of Caragana tibetica nibbling. * represents p<0.05; ** represents p<0.01.

### 3.3 Changes of osmoregulatory substances in *Caragana tibetica* under different simulated nibbling intensities

During the growing season from May to September, different simulated nibbling intensities had significant effects on the PRO content of *Caragana tibetica*. As shown in **Table 3**, the 75% nibbling intensity significantly increased by 22.93% compared with the 50% nibbling intensity in May (*p*<0.05). In June and August, PRO content showed a significant increasing trend with the increase of nibbling intensity, while in July, 50% and 75% nibbling intensity were significantly greater than CK and 25%. Among May to September, the PRO content of all treatment groups in July was higher than that of other groups, the highest value was 180.82 μg•g^-1^ at 75% nibbling intensity, and the lowest value was 119.72 μg•g^-1^ for CK. From the table, it can be seen that SS and SP content increased first and then decreased during the whole growing season, reaching the maximum value in July. SS content was between 3.34 mg•g^-1^ and 4.61 mg•g^-1^ in July, and was significantly increased by 17.37% and 27.55% compared with CK in 50% and 75% treatment groups, respectively (*p*<0.05). In May, 75% nibbling intensity was larger than in other groups, a significant increase of 16.2% compared with CK. SP content in July and August was higher than in other treatment groups. From June to August, SP content increased with the increase in nibbling intensity. In August, there was no significant difference in SP content among 25%, 50% and 75% treatment groups (*p*>0.05). In summary, PRO, SS, and SP contents reflect the physiological response and adaptability of *Caragana tibetica* in the growing season.

**Table 3:** Changes of three osmoregulatory substances content in *Caragana tibetica* leaves under different simulated nibbling intensities.

Month	May				June				July				August				September			
SGI	CK	25%	50%	75%	CK	25%	50%	75%	CK	25%	50%	75%	CK	25%	50%	75%	CK	25%	50%	75%
PRO/ μg•g^-1^	53.16±2.67bc	56.72±1.33ab	50.46±4.43c	62.03±3.08a	68.49±1.71d	75.72±1.46c	82.25±2.7b	88.2±3.1a	119.72±7.37b	134.5±22.13b	159.15±5.51a	180.82±4.69a	88.59±1.47d	96.91±3.05c	112.33±2.78b	139.88±3.97a	50.88±2.88c	55.26±0.41b	58.49±2.27b	76.48±0.79a
SS/ mg•g^-1^	1.76±0.08c	1.80±0.10ab	1.89±0.03bc	2.05± 0.05a	1.82±0.14c	2.15± 0.16b	2.29±0.05a	2.94±0.15a	3.34±0.14c	3.53±0.12c	3.92±0.14b	4.61±0.27a	2.70±0.08c	3.18±0.20b	3.62± 0.08a	3.84±0.13a	1.73±0.04d	1.95±0.18c	2.19±0.05b	2.84±0.07a
SP/ mg•g^-1^	1.19± 0.06c	1.34±0.03ab	1.23±0.11bc	1.37±0.03a	1.66± 0.11c	1.87±0.02b	2.06±0.05a	2.15±0.02a	2.26± 0.05c	2.53±0.11b	2.62±0.13b	2.84±0.06a	2.06± 0.06b	2.53±0.26a	2.65± 0.11a	2.75±0.12a	1.37±0.21bc	1.50±0.07b	1.23±0.03c	1.90±0.08a

Notes: Comparisons were made within the same month group under the same indicator, and there were no significant differences between data with the same lowercase letter (p<0.05). SGI represents simulated nibbling intensities.

### 3.4 Changes of MDA in *Caragana tibetica* under different simulated nibbling intensities

MDA content reflects the physiological state and adaptive ability of *Caragana tibetica*. As shown in **Table 4**, under different nibbling intensities, the MDA content of *Caragana tibetica* increased first and then decreased from May to September, reaching the maximum value in July. The values were 3.34 μmol•g^-1^、3.53 μmol•g^-1^、3.92 μmol•g^-1,^ and 4.61μmol•g^-1^ at CK, 25%, 50%, and 75% nibbling intensity, respectively, while MDA in 50% and 75% treatment groups was significantly higher than that in CK and 25% (*p*<0.05). MDA content was the lowest in May, at CK, 25%, 50%, and 75%, the values were 1.76 μmol•g^-1^, 1.80 μmol•g^-1^, 1.89 μmol•g^-1,^ and 2.05 μmol•g^-1^, respectively, while 75% nibbling intensity was significantly higher than that of the other three groups (*p*<0.05). Besides, there was no significant difference between CK and 25% in May, June, August, and September, while the nibbling intensity of 25% in July was significantly higher than that of CK. In summary, the MDA content increased with the increase of nibbling intensity in each month of the whole growing season, indicating that the greater the nibbling intensity, the greater the effect on the MDA content of *Caragana tibetica*.

**Table 4:** Changes of MDA content in *Caragana tibetica leaves* under different simulated nibbling intensities.

Month	May				June				July				August				September			
SGI	CK	25%	50%	75%	CK	25%	50%	75%	CK	25%	50%	75%	CK	25%	50%	75%	CK	25%	50%	75%
MDA/ μmol•g^-1^	1.76±0.07b	1.80±0.05ab	1.89±0.06b	2.05±0.04a	1.48±0.11b	1.47±0.17b	1.81±0.02a	1.84±0.11a	3.34±0.03c	3.53±0.13b	3.92±0.06a	4.61±0.07a	2.35±0.12b	2.52±0.1b	2.51±0.07b	2.83±0.08a	1.81±0.06c	1.92±0.07c	2.09±0.06b	2.29±0.11a

Notes: Different letters indicated that there were significant differences in MDA under different simulated nibbling intensities (p<0.05). SGI represents simulated nibbling intensities.

### 3.5 Correlation analysis of growth and physiological characteristics of *Caragana tibetica* after simulated nibbling

As shown in **Figure 1**, NBN and NBL were positively correlated with SP, MDA, PRO, SOD, POD and CAT, but not significantly. There was a significant positive correlation between NBL and SS (*p*<0.05). SS, SP, MDA, PRO, SOD, POD and CAT were significantly positively correlated with each other (*p*<0.01).

## 4 Discussion

### 4.1 Growth characteristics of *Caragana tibetica* under different nibbling intensities

From the simulated nibbling experiment, it is clear that the nibbling treatment had an important effect on the growth performance of *Caragana tibetica*. After nibbling treatment, NBN and NBL of *Caragana tibetica* were significantly higher than those of the control group. Under different nibbling intensities, NBN and NBL showed the same trend, i.e., 75% nibbling>50% nibbling>25% nibbling>CK. This result may be due to compensatory growth mechanisms of *Caragana tibetica*. When the branches and leaves of *Caragana tibetica* are damaged, they may reduce the normal leaf fall or increase the photosynthetic rate of the *Caragana tibetica* to compensate for the nibbling loss. This compensatory growth mechanism helps *Caragana tibetica* recover quickly after injury and may even grow better than if it were undamaged. These results are consistent with the description of compensatory growth mechanisms and their ecological significance after nibbling or injury in Fornoni (2011).

### 4.2 Physiological characteristics of *Caragana tibetica* under different nibbling intensities

From the experiment, it can be found that the status of the active oxygen metabolism system in *Caragana tibetica* changed under different nibbling intensities. This change causes the protective enzyme system in the *Caragana tibetica* to be activated and participate in the clearance process of reactive oxygen species. The activity of antioxidant enzymes such as POD, SOD, and CAT in *Caragana tibetica* changes with the nibbling intensity. The results showed that during the whole growing season from May to September, the main protective enzymes such as POD, SOD, and CAT showed similar changes under different nibbling intensity. This tendency is usually manifested as an initial increase in growth (May to July) followed by a gradual decrease (July to September). This is consistent with the results of Zhu et al. (2011). Considering that large amounts of antioxidant enzymes are usually synthesized to protect cells from oxidative damage, maintaining the antioxidant enzyme activity in *Caragana tibetica* at a high level can help maintain its normal growth and development.

In addition, the results showed that osmoregulatory substances play an important role in the *Caragana tibetica* stress response. As an important amino acid, PRO can play functions such as anti-oxidation, anti-stress, and regulation of growth and development in *Caragana tibetica*. It was found that under different nibbling treatments, *Caragana tibetica* would synthesize more PRO to cope with external pressure with the increase of months. In the same month, the PRO content produced by *Caragana tibetica* under 75% nibbling treatment was significantly higher than CK, while there was no significant difference from 50% nibbling treatment in some months. Furthermore, this paper found that SS and SP show similar changes to PRO, which indicates that under nibbling treatment, SS and SP can also regulate osmotic regulation and cell protection to maintain cell stability. It was found that the contents of PRO, SS, and SP in *Caragana tibetica* were significantly affected by different nibbling intensities. Specifically, from May to September, the contents of three types of osmoregulatory substances increased first and then decreased, and reached their highest in July of the 75% nibbling treatment. The results indicate that 75% nibbling treatment may promote the ability of *Caragana tibetica* to adapt to external stress, while 50% and 25% nibbling treatment have a higher effect on membrane lipid permeability. The accumulation of PRO and stress response, the physiological function of SS and the role of SP in this paper are consistent with the conclusions of Lehmann et al. (2010).

As for MDA, its content level can reflect the degree of stress injury and the degree of membrane lipid peroxidation. In this paper, the results showed that the maximum MDA content under different nibbling intensity treatments was 1.19 times, 1.33 times, and 1.36 times that of the control treatment, respectively, which reflects that 75% nibbling intensity had the greatest effect on the biofilm lipid permeability of *Caragana tibetica*. With the progress of the growing season (from May to September), MDA content increased first and then decreased, which also reached its maximum value in July. In September, there were significant differences between 50% & 75% nibbling treatments and CK, while the difference between 75% nibbling treatments and other nibbling treatments is also remarkable. It showed that higher nibbling intensity may lead to greater stress in *Caragana tibetica*, thus intensifying oxidation and lipid peroxidation in leaves. In addition, nibbling pressure can lead to the intensification of membrane lipid peroxidation, which is manifested by the increase of MDA content. This reflects the degree of damage plant cell membrane, suggesting a change in cell membrane permeability and the impairment of physiological function in the face of nibbling pressure. The research results in this paper are consistent with the significance of MDA as a marker of membrane lipid peroxidation proposed by Davey et al (2005) and its change rule in plant stress response.

## 5 Conclusion

NBN and NBL under three different nibbling intensities were greater than those in CK. NBN and NBL increased with higher nibbling intensity, which indicates that nibbling can promote the growth of new branches of *Caragana tibetica*. Different simulated nibbling intensities had effects on the antioxidant enzyme activity of *Caragana tibetica*. The contents of POD, SOD and CAT reached highest in July during the growth season from May to September. Different simulated nibbling intensities had significant effects on the PRO content of *Caragana tibetica*. During the whole growing season, the contents of SS and SP increased first and then decreased, reaching the maximum value in July. MDA content increased with the nibbling intensity rise of the whole growing season, indicating that the greater the nibbling intensity, the greater the effect on the MDA content of *Caragana tibetica*. In future work, we need to further employ metabolomics techniques to reveal the effects of simulated nibbling on aspects of *Caragana tibetica* gene regulation.

## Data Availability

The original contributions presented in the study are included in the article/supplementary material. Further inquiries can be directed to the corresponding author.
